# Promoting Cancer Screening in Partnership With Health Ministries in 9 African American Churches in South Los Angeles: An Implementation Pilot Study

**DOI:** 10.5888/pcd16.190135

**Published:** 2019-09-19

**Authors:** Annette E. Maxwell, Aziza Lucas-Wright, Rhonda E. Santifer, Claudia Vargas, Juana Gatson, L. Cindy Chang

**Affiliations:** 1 Center for Cancer Prevention and Control Research, University of California, Los Angeles Fielding School of Public Health and Jonsson Comprehensive Cancer Center, Los Angeles, California; 2 Division of Cancer Research and Training, Charles R. Drew University of Medicine and Science, Los Angeles, California

## Abstract

**Purpose and Objectives:**

We conducted a pilot study to assess the degree to which an intervention led by community health advisors (CHAs) to promote cancer screening was delivered as intended and to estimate the potential effect of the intervention on receipt of screening. In contrast to previous studies and to maximize its potential public health impact, the intervention targeted 4 screening tests and only participants who were not up to date with screening guidelines for at least 1 cancer. Because CHAs had to both determine baseline adherence and provide counseling on 4 screening tests, the protocol was complex. Complex protocols can reduce implementation fidelity.

**Intervention Approach:**

In partnership with health ministries at 9 African American churches in South Los Angeles, we conducted a 1-group pretest–posttest pilot study to assess the feasibility of implementing the intervention. CHAs recruited and obtained consent from church members aged 50 to 75 years; assessed adherence to national screening guidelines for breast, cervical, colorectal, and prostate cancer; and provided evidence-based strategies (one-on-one counseling, print materials, reminder calls) to encourage screening for tests that were overdue.

**Evaluation Methods:**

We assessed implementation fidelity by reviewing baseline screening assessments and counseling scripts completed by CHAs. We estimated potential effect of the intervention on receipt of screening by using data from 3-month follow-up surveys, conducted by the research team, of participants who were nonadherent at baseline.

**Results:**

From June 2016 to June 2018, 44 CHAs conducted baseline assessments of 775 participants, of whom 338 (44%) were nonadherent to national guidelines for 1 or more cancer screening tests. CHAs provided counseling to most nonadherent participants. At follow-up, about one-third of participants reported that they had discussed cancer screening with their provider and a smaller proportion reported receipt of a screening test; 13% of men and 25% of women reported receipt of colorectal cancer screening.

**Implications for Public Health:**

This study demonstrates that with training and ongoing technical assistance, CHAs at African American health ministries can implement complex research protocols with good fidelity.

**Summary**

**What is already known on this topic?**

Church–academic partnerships hold promise for reducing cancer disparities, and church-based programs are feasible and acceptable in many populations, including African Americans.

**What is added by this report?**

We examined the extent to which trained community health advisors (CHAs) implemented a complex intervention that included assessing adherence to screening guidelines and counseling for up to for 4 cancers.

**What are the implications for public health practice?**

Counseling for multiple cancer screening tests can increase the potential public health impact of the intervention. However, complex protocols can reduce implementation fidelity. This study demonstrates that with training and ongoing technical assistance, CHAs at African American health ministries can implement complex research protocols with good fidelity.

## Introduction

African Americans have a disproportionate burden of cancer. They are more likely to develop and die of cancer than members of any other racial or ethnic group. Leading causes of cancer death among African Americans are lung, colorectal, breast, and prostate cancer (1). Cancer screening is effective in reducing the burden of breast, cervical, and colorectal cancer. The US Preventive Services Task Force recommends screening for colorectal cancer by using fecal occult blood testing, sigmoidoscopy, or colonoscopy for adults aged 50 to 75 (2); biennial mammography for women aged 50 to 74 (3); and screening for cervical cancer for women aged 21 to 65 with a Papanicolaou (Pap) test every 3 years or, for women aged 30 to 65 who want to lengthen the screening interval, screening with a combination of a Pap test and human papillomavirus test every 5 years (4). African American men have the highest mortality for prostate cancer of any racial or ethnic group in the United States (1). The US Preventive Services Task Force recommends that men discuss the potential benefits and harms of prostate-specific antigen (PSA) testing with their physicians so they can make an informed decision whether or not they want to get tested (5). To reduce cancer disparities, these recommendations for screening and informed decision making need to be widely implemented.

Church–academic partnerships hold promise for reducing cancer disparities (6) and church-based programs are feasible and acceptable in many racial/ethnic minority populations, including African Americans (7–10). Many churches have health ministries that are dedicated to improving the overall health of their members. In the African American community, which has been marginalized and mistreated in biomedical research, churches are trusted sources of information and support. Churches often conduct health programs through community health advisors (CHAs), trained lay people who are well known and respected by other church members. CHAs can serve as health advisors, referral sources, and role models in addition to distributing materials and advocating on behalf of community members. With training, they can also recruit participants into health studies, implement programs, document their activities, and collect data (8,11–15).

Several interventions have promoted cancer screening in collaboration with African American churches. Although most interventions promoted screening for only 1 cancer site (16–19), a recent intervention promoted multiple cancer screening tests (10). In addition, previous studies included participants regardless of their baseline screening status (10,17) and observed that the intervention had a limited opportunity to affect screening rates if screening rates were high in the study sample at baseline. To our knowledge, only 1 previous study focused on church members not up to date with colorectal cancer screening; in that study, the research team identified eligible participants through a baseline survey (19). Intervention strategies have included small group sessions (10,17), distribution of print materials (17,20), tailored newsletters (19), and individual counseling and reminder calls (19,21). Many of these strategies are recommended by the Community Guide (www.thecommunityguide.org).

Given this body of literature and to maximize the potential public health impact of our intervention, we decided to partner with health ministries at African American churches to promote cancer screening for multiple sites (breast, cervix, prostate, and colon/rectum) and to focus our efforts on church members aged 50 to 75 who were nonadherent to the national cancer screening guidelines for at least 1 of these sites. As part of capacity building efforts, we trained CHAs to assess adherence to cancer screening guidelines rather than having the research team conduct the assessment.

## Purpose and Objectives

This article describes the pilot implementation of a protocol by trained CHAs. The protocol consisted of 2 main components: 1) a baseline assessment to determine adherence to screening guidelines for multiple cancers and 2) the implementation of up to 3 intervention strategies to promote adherence to guidelines that were not met. Compared with previous research protocols in African American churches (10,17,19), our research protocol was complex: CHAs had to become familiar with screening tests and screening guidelines for breast, cervical, colorectal, and prostate cancer to identify nonadherent persons and to provide counseling on 1 or more screening tests, as needed.

Implementation fidelity is critical to successful translations of evidence-based strategies into practice (22), but fidelity may be reduced if the intervention is complex and difficult to learn (23). We conducted a pilot study to assess the degree to which this protocol was delivered as intended and to estimate the potential effect of the intervention on receipt of screening.

## Intervention Approach

**Figure 1:**
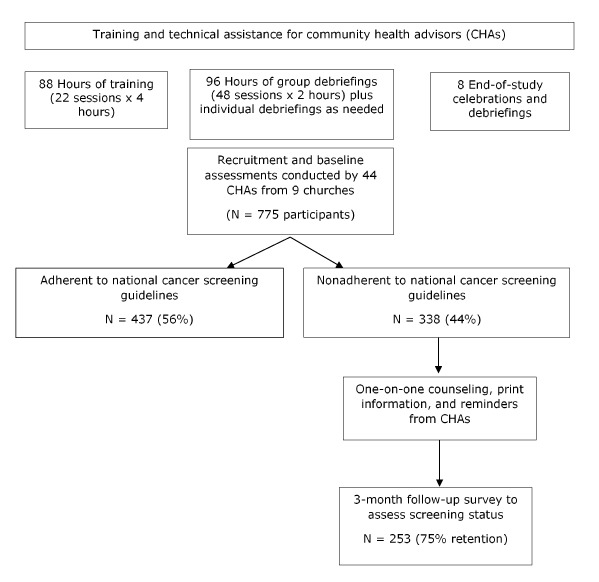
One-group pretest–posttest design of study to promote screening for 4 types of cancer (breast, cervical, colorectal, and prostate) among members of 9 African American churches in South Los Angeles, 2016–2018.

In partnership with 9 churches in South Los Angeles, we conducted a 1-group pretest–posttest pilot study (Figure 1) from June 2016 to June 2018. Trained CHAs from each church recruited participants aged 50 to 75 and assessed their adherence to cancer screening guidelines. CHAs promoted cancer screening for eligible participants who were not adherent to national cancer screening guidelines for breast, cervical, or colorectal cancer. They also encouraged men to discuss the value of prostate cancer screening with a physician (24). The study protocol and all assessment instruments were approved by the institutional review board of the University of California, Los Angeles.

Similar to other health promotion studies in African American churches (19,20), our CHA-led intervention primarily used individually directed evidence-based strategies to promote cancer screening. Intervention components were one-on-one education and counseling using a script, distribution of print information, and reminder telephone calls. The intervention approach was further informed by a previous needs assessment among 800 members of African American churches in South Los Angeles (25), who indicated that hearing about cancer prevention and screening at church would be very helpful (83%) or somewhat helpful (12%) and that they liked to receive information from trained peers (77%). Studies show that CHAs can promote cancer screening, especially if they are matched to participants on race or ethnicity (26–28). The previous needs assessment also found that the main barriers for not obtaining cancer screening tests were “never thought about it,” “doctor did not tell me I needed it,” and “put it off” (25). This information suggests the need for reminders and barrier counseling strategies in this community. We decided to offer one-on-one counseling rather than small group educational sessions at church because other studies showed that only a minority of study participants attend multiple workshops (10) and given the choice, many CHAs prefer to provide one-on-one counseling, which does not require scheduling of a group event (9).

Our intervention approach is consistent with the Social Ecological Framework, which recognizes that individuals are part of families that belong to organizations and social groups, all of which can influence health behavior (29,30). Although most of the intervention was directed at individuals, we also encouraged CHAs to plan a few activities to promote cancer screening at the church level during the initial training session.

### Recruitment of churches and community health advisors

Leveraging strong ties of research team members to African American Christian churches in South Los Angeles, we invited 11 churches to partner with us in a study to promote cancer screening. All churches agreed to participate and identified 5 (and in one larger church 9) people to serve as a CHA. Each CHA was asked to recruit at least 10 participants, to assess their screening status, and to counsel those who were not up to date with screening. Two of the 11 churches dropped out immediately after the training because of competing activities. Each of the remaining 9 churches committed to partner with us for 12 months and received a $2,000 stipend. Each CHA received up to $500 in stipends to incentivize their participation.

### Training and technical assistance provided to CHAs

We provided two 4-hour training workshops for CHAs at each church to familiarize CHAs with the study protocol, cancer risk factors and screening guidelines, and the rules governing research with human participants. More details on the training and the CHAs and an evaluation of the training are described elsewhere (31). By using demonstration and role play, CHAs practiced obtaining consent from potential participants; administering a 1-page baseline assessment to assess screening for breast and cervical cancer (women only), PSA testing and discussion (men only), and colorectal cancer screening; and using evidence-based strategies to promote screening. CHAs were also provided with a list of low-cost or free programs to which they could refer participants without health insurance.

Each church team discussed strategies to inform people attending their church about the study and to promote screening churchwide. With guidance from the research team, each team of CHAs developed SMART objectives (specific, measurable, achievable, realistic, time-bound) for the next 6 to 12 months to promote cancer screening. SMART objectives centered on publishing personal interest stories (eg, testimonials of cancer survivors); organizing a worship service that featured a health theme in the sermon or dedicating 1 Sunday per month to health promotion; and making presentations to inform church members and visitors about the study. An example of a SMART objective is “The Health Council will organize a 15-minute talk on Father’s Day (June 19, 2016) by [name of presenter] to inform parishioners about the study and to introduce the CHAs to the congregation.”

After completion of the 2 training workshops, the research team conducted debriefing sessions with CHAs at each church every 6 to 8 weeks to answer questions, collect completed study documents (baseline surveys and counseling scripts with CHA notes), and provide CHAs with additional study materials, as needed (Figure 1).

### Implementation of the CHA-delivered intervention

To assist CHAs in implementing the one-on-one education in a standardized fashion, we provided a 5-part counseling script for each cancer site (breast, cervix, colon/rectum, prostate).

**Part 1.** Each script started with an interesting fact pertaining to the African American community to raise interest in the topic and to explain its importance. For example:

Among cancers that affect both men and women, colorectal cancer is the second leading cancer killer in the US. According to studies, African Americans are at a higher risk for the disease than other populations. Research shows that African Americans are being diagnosed at a younger age and death rates from colorectal cancer are higher among African Americans than any other group in the United States.

**Part 2.** Next, CHAs asked if the participant ever had a particular screening test and explained each test, if needed, using print materials from the National Cancer Institute or the American Cancer Society. The materials included pictures of the test.

**Part 3.** Counseling on barriers to screening started with an open-ended question about any reasons why the participant did not have the test according to the guidelines. CHAs checked off the barriers that were mentioned and probed for other barriers that were not mentioned. For each barrier, a short response was provided for CHAs to use during the counseling. For example, if the barrier was “no time,” the CHA responded with, “It is important to make time for your health. The things you are putting off now because you ‘don’t have time’ are going to cost you much more than time if you wait until it is too late.”

**Part 4.** In this part, the goal-setting section, the CHA asked participants if they were ready to ask their doctor about getting the cancer screening test that was overdue. CHAs also asked respondents to set a goal for the time frame.

**Part 5.** The conversation ended with the CHA asking for permission to call again in 3 or 4 weeks to see if the participant had more questions or needed more help in getting the test.

## Evaluation Methods

We selected evaluation methods to assess implementation fidelity, the acceptability of the CHA-led intervention, and the potential effect on cancer screening promotion. We developed brief assessments, keeping in mind the burden they posed to CHAs and respondents.

### Measures

**Figure 2:**
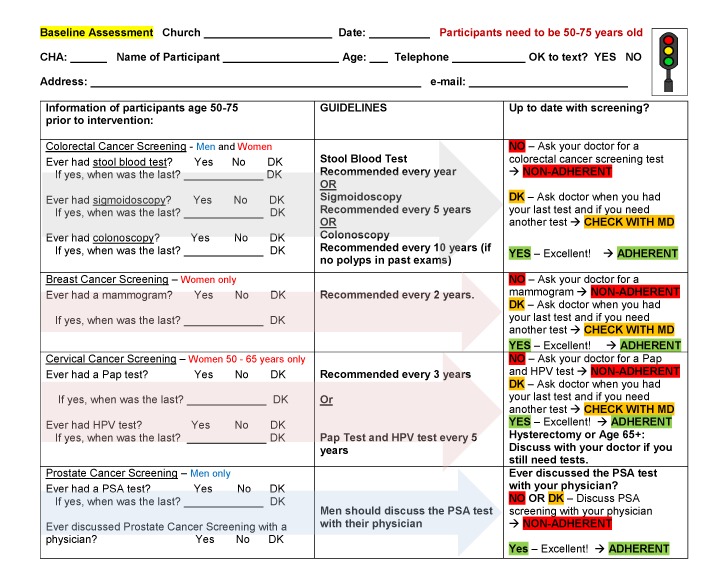
One-page baseline questionnaire used by community health advisors to assess adherence to cancer screening guidelines in 9 African American churches participating in an intervention in Los Angeles, 2016–2018. Abbreviations: DK, don’t know; HPV, human papilloma virus; MD, doctor; Pap, Papanicolaou; PSA, prostate specific antigen.

The 1-page baseline assessment asked for name, age, address, and telephone number of the participant and whether the participant had received any of the following: stool blood test, sigmoidoscopy, colonoscopy; mammogram, Pap test, human papilloma virus test (women only); and PSA test (men only, ever and when most recent) (Figure 2). In addition, the assessment asked men if they had discussed the value of PSA testing with a physician (ever). Color coding indicated questions only asked of women and only asked of men. CHAs compared the screening history of each participant to the guidelines that were also provided on the form, and informed participants if they were adherent to the national cancer screening guidelines and what test(s) they needed to request from their physician, if any. Traffic light color coding was used to assist CHAs in determining if a participant was nonadherent (highlighted in red), adherent (highlighted in green), or had to check with the doctor (highlighted in yellow) to determine if they needed a screening test. Short explanations of each screening test were provided for CHAs to read to participants, if needed. To keep the baseline assessment brief, no further information was asked of participants. The research team reviewed completed baseline assessments with CHAs during debriefing meetings at each church every 6 to 8 weeks to answer questions and to ensure that each participant was correctly classified as “adherent with *all* recommended cancer screening guidelines” or “not adherent with at least *one* recommended cancer screening guideline.”

**Table:** 

Screening Type	Guideline	Up to Date With Screening?
**Colorectal cancer screening (men and women)**		
Ever had stool blood test? Answers are yes, no, or don’t know. If yes, when was the last? [Fill in date] or don’t know.	Stool blood test recommended every year or sigmoidoscopy recommended every 5 years or colonoscopy recommended every 10 years (if no polyps in past exams)	No [in red]: Ask your doctor for a colorectal cancer screening test. Marked as nonadherent [in red]. Don’t know [in yellow]: Ask doctor when you had your last test and if you need another test. Marked as “Check with MD” [in yellow]. Yes [in green]: Excellent! Marked as adherent [in green].
Ever had sigmoidoscopy? Answers are yes, no, or don’t know. If yes, when was the last? [Fill in date] or don’t know.		
Ever had colonoscopy? Answers are yes, no, or don’t know. If yes, when was the last? [Fill in date] or don’t know.		
**Breast cancer screening (women only)**		
Ever had a mammogram? Answers are yes, no, or don’t know. If yes, when was the last? [Fill in date] or don’t know.	Recommended every 2 years	No [in red]: Ask your doctor for a mammogram. Marked as nonadherent [in red]. Don’t know [in yellow]: Ask doctor when you had your last test and if you need another test. Marked as “Check with MD” [in yellow]. Yes [in green]: Excellent! Marked as adherent [in green].
**Cervical cancer screening (women 50–65 years only)**		
Ever had a Pap test? Answers are yes, no, or don’t know. If yes, when was the last? [Fill in date] or don’t know.	Recommended every 3 years or Pap test and HPV test every 5 years	No [in red]: Ask your doctor for a Pap and HPV test. Marked as nonadherent [in red]. Don’t know [in yellow]: Ask doctor when you had your last test and if you need another test. Marked as “Check with MD” [in yellow]. Yes [in green]: Excellent! Marked as adherent [in green]. Hysterectomy or age ≥65: Discuss with your doctor if you still need tests.
Ever had HPV test? Answers are yes, no, or don’t know. If yes, when was the last? [Fill in date] or don’t know.		
**Prostate cancer screening (men only)**		
Ever had a PSA test? Answers are yes, no, or don’t know. If yes, when was the last? [Fill in date] or don’t know.	Men should discuss the PSA test with their physician	Ever discussed the PSA test with your physician? Answers of no [in red] or don’t know [in yellow]: Discuss PSA screening with your physician. Marked as nonadherent [in red]. Yes [in green]: Excellent! Marked as adherent [in green].
Ever discussed prostate cancer screening with a physician? Answers are yes, no, or don’t know.		

**Implementation fidelity.** CHAs documented their counseling efforts with each participant in the notes section of the counseling script, including the barrier they discussed and whether or not they distributed print information and issued another reminder 3 or 4 weeks after the initial counseling session. Implementation fidelity (receipt of counseling, coded as yes or no) was also assessed in the 3-month follow-up survey of study participants.

**Three-month follow-up survey of participants who were nonadherent at baseline**. Participants who were nonadherent at baseline to at least 1 screening test were contacted again for a 3-month follow-up survey. This survey assessed receipt of needed screening test(s) and discussion of cancer screening with a health care provider during the follow-up period (both of these outcomes were encouraged by CHAs); receipt of intervention components (fidelity of intervention implementation as reported by participants); and satisfaction with the educational session and information on demographic characteristics and access to care. The 3-month survey was conducted by telephone by 1 member of the research team who was not involved in the intervention, rather than by multiple CHAs. This reduced social desirability bias, decreased the workload for CHAs, and facilitated a more consistent administration of the survey. The follow-up survey took 20 minutes on average. Participants who completed the 3-month follow-up survey received a $20 store gift card.

**Twelve-month debriefing of CHAs.** The debriefings were conducted during the end-of-study meeting and celebration at 8 churches. During this 2-hour meeting, we discussed implementation of each SMART objective and barriers to implementation and presented certificates of participation to CHAs; we then hosted a catered lunch to show our appreciation.

### Statistical analysis

We conducted all analyses by using SAS 9.4 (SAS Institute Inc). We used descriptive statistics to describe demographic information and access to care and receipt of intervention components. We compared the sex, age, zip code, and baseline screening rates of participants who completed the 3-month follow-up with the characteristics of those who did not. We also compared the characteristics of men and women who completed the 3-month follow-up survey. Both comparisons used χ^2^ tests for categorical variables and 2-sample *t* tests for continuous variables.

We assessed the potential effect size of the intervention for 2 outcomes: 1) discussion of cancer screening with a provider during the follow-up period (asked of all participants at posttest) and 2) receipt of a specified screening test during the follow-up period (asked only of participants nonadherent at baseline who needed a specific test). We assessed these 2 outcomes in 2 ways: “as reported” by those who completed the 3-month follow-up survey and as a “worst-case” scenario. The worst-case scenario included all participants who completed the baseline assessment; in this scenario, missing information in the follow-up survey was coded as “not discussed” or “not screened.” Analyses on discussion and receipt of a Pap test included only women aged 50 to 65 who did not report having had a hysterectomy.

## Results

During the study period, 44 CHAs from 9 African American churches conducted baseline assessments of 775 participants, of whom 338 (44%) were nonadherent to national screening guidelines for 1 or more cancer screening tests. More than 90% of baseline assessments and counseling sessions were conducted face-to-face and the rest by telephone. Of the 338 participants who were nonadherent at baseline, 253 completed follow-up telephone surveys, for a retention rate of 75% (Figure 1). Only 20 participants (6%) refused to complete the follow-up survey. In addition, 19% of participants were lost to follow-up because of disconnected or wrong telephone numbers or because they never answered the telephone.

### Implementation of SMART objectives by participating churches

Churches developed 4 to 8 SMART objectives (Table 1). One church with 4 SMART objectives completely implemented all of them. Most churches with a larger number of SMART objectives completely implemented at least 4 of them. Activities that were completely implemented at several churches included development of cancer awareness buttons or T-shirts that said, “I am a Community Health Advisor. Ask me!”; study announcements by the pastor; addition of information on cancer screening in the church bulletin; recognition of cancer survivors in the congregation; and testimonials related to cancer or cancer screening.

**Table 1:** Implementation of SMART Objectives by 9 Participating African American Churches, According to Data From 12-Month Debriefings of CHAs, Los Angeles, 2016–2018^a^

Church	No. of SMART Objectives Proposed by CHAs	Implementation of SMART Objectives at 12-Month Follow-Up		
		Completely Implemented, No.	Partially Implemented, No.	Not Implemented, No.
A	4	4	0	0
B	4	1	2	1
C	5	4	1	0
D	5	—b	—b	—b
E	6	4	1	1
F	6	4	1	1
G	7	5	1	1
H	7	4	1	2
I	8	4	2	2

Abbreviations: CHA, community health advisor; SMART, specific, measurable, achievable, realistic, time-bound.

a Data were collected in a 1-group pretest–posttest pilot study to assess the feasibility of implementing an intervention led by CHAs to promote cancer screening (for breast, cervical, colorectal, and prostate cancer) at 9 African American churches in South Los Angeles.

b Church did not complete the 12-month debriefing.

Barriers to implementation included being too busy with other activities, having a small congregation and not enough volunteers to support planned activities (eg, a quarterly mini health fair), discontinuing an activity because it was not successful (eg, having a regular table with sign-up sheets after services), and choosing an activity that sounded exciting and creative but was too difficult to implement (eg, a skit with the dance ministry).

### Implementation of intervention components by CHAs

According to completed counseling scripts, CHAs provided counseling on 1) colorectal cancer screening to 185 of 226 participants who were overdue, 2) breast cancer screening to 77 of 110 women, 3) cervical cancer screening to 48 of 60 women aged 50 to 65 years, and 4) PSA informed decision making to 74 out of 102 men (Table 2). The barriers to screening that were most frequently mentioned by participants and that were addressed during the counseling were “no time” (up to 51% among women nonadherent to breast cancer screening), “I feel fine,” and “I don’t like to go to doctors.” Barriers that were frequently mentioned for specific screening tests included “Don’t like preparation or procedure for colonoscopy” (20%), “pain or discomfort of mammogram” (9%) and “did not know about the PSA test” (41%). CHAs distributed print materials on screening tests during more than half of the counseling sessions. They issued a follow-up reminder, usually a telephone call, for about 40% of the counseling sessions.

**Table 2:** Implementation of 3 Intervention Strategies by CHAs Among Intervention Participants Who Were Nonadherent to ≥1 Cancer Screening Guideline at Baseline, According to Counseling Scripts^a^ Completed by CHAs, Los Angeles, 2016–2018^b^

Strategy	Colorectal Cancer Screening	Breast Cancer Screening	Cervical Cancer Screening^c^	PSA Discussion^d^
**Conducted one-on-one counseling to discuss barriers**	185 of 226 (82)	77 of 110 (70)	48 of 60 (80)	74 of 102 (73)
Barriers specified by participants				
No time	85 of 185 (46)	39 of 77 (51)	20 of 48 (42)	29 of 74 (39)
I feel fine	48 of 185 (26)	14 of 77 (18)	7 of 48 (15)	19 of 74 (26)
Don’t like going to doctors	33 of 185 (18)	12 of 77 (16)	6 of 48 (12)	18 of 74 (24)
No insurance	13 of 185 (7)	8 of 77 (10)	6 of 48 (12)	6 of 74 (8)
No transportation	3 of 185 (2)	3 of 77 (4)	2 of 48 (4)	2 of 74 (3)
Do not like preparation or procedure for colonoscopy	37 of 185 (20)	—e	—e	—e
Cannot take day off work for colonoscopy	10 of 185 (5)	—e	—e	—e
Do not have someone to drive me to colonoscopy	7 of 185 (4)	—e	—e	—e
Do not like to handle stool	11 of 185 (6)	—e	—e	—e
Do not know how to do stool blood test	4 of 185 (2)	—e	—e	—e
Pain or discomfort of mammogram	—e	7 of 77 (9)	—e	—e
Fear of being diagnosed with breast cancer	—e	5 of 77 (6)	—e	—e
Don’t like the preparation or procedure for Pap test	—e	—e	3 of 48 (6)	—e
Did not know about PSA test	—e	—e	—e	30 of 74 (41)
**Distributed print materials**	114 of 185 (62)	42 of 77 (55)	28 of 48 (58)	48 of 74 (65)
**Issued a follow-up reminder**	86 of 185 (46)	31 of 77 (40)	20 of 48 (42)	31 of 74 (42)

Abbreviations: CHA, community health advisor; Pap, Papanicolaou; PSA, prostate-specific antigen.

a To assist CHAs in implementing the one-on-one education in a standardized fashion, we provided a 5-part counseling script for each cancer site (breast, cervix, colon/rectum, prostate). CHAs documented their counseling sessions with each participant in the notes section of the counseling script.

b Data were collected in a 1-group pretest–posttest pilot study to assess the feasibility of implementing an intervention led by CHAs to promote cancer screening (for breast, cervical, colorectal, and prostate cancer) at 9 African American churches in South Los Angeles. All values are numerator and denominator (percentage).

c Only includes 60 women aged 50 to 65 without a hysterectomy who were not adherent to cervical cancer screening guidelines at baseline.

d The US Preventive Services Task Force recommends that men discuss the potential benefits and harms of PSA testing with their physician so they can make an informed decision whether or not they want to get tested (5).

e Does not apply.

According to 3-month follow-up surveys, most participants said that they had discussed their cancer screening history and which screening test they needed with a CHA (80%), that the CHA had recommended they discuss cancer screening with their physician (91%) and obtain a cancer screening test (72%), and that the CHA provided print materials on cancer screening (65%). These data, provided by participants, were consistent with the implementation data derived from counseling scripts completed by CHAs.

Although 44 CHAs performed baseline assessments, only 38 CHAs provided counseling, according to counseling scripts: 11 CHAs performed counseling for 1 to 4 overdue screening tests, 13 CHAs performed counseling for 5 to 10 overdue screening tests, and 14 of 44 CHAs performed counseling for more than 10 overdue screening tests.

### Characteristics of participants

Of the 338 participants who were nonadherent at baseline to at least 1 national cancer screening guideline, 124 were men and 214 were women aged 50 to 75. Of the 253 participants who completed the 3-month follow-up survey, 95 were men and 158 were women aged 50 to 75; mean age was approximately 60, and education ranged from less than high school graduate to college graduate (Table 3). Women were significantly more likely than men to have private health insurance (*P* = .001), to have a regular doctor (*P* < .001), to ever go to a physician for a medical checkup (*P* < .001), and to have had a routine checkup in the past 12 months (*P* = .03).

**Table 3:** Characteristics of Participants Who Were Nonadherent to ≥1 Cancer Screening Guideline at Baseline and Completed the 3-Month Follow-Up Survey (N = 253), Los Angeles, 2016–2018^a^

Characteristic	Men (N = 95)	Women (N = 158)	*P* Value^b^
**Age, mean (SD) [range], y**	58.8 (6.0) [50–75]	60.3 (7.1) [50–75]	.08
**Residence**			
South Los Angeles zip code	58 of 95 (61)	87 of 158 (55)	.35
Other zip code	37 of 95 (39)	71 of 158 (45)	
**Marital status**			
Single	45 of 95 (47)	54 of 157 (34)	.08
Married	30 of 95 (32)	53 of 157 (34)	
Divorced, separated, or widowed	20 of 95 (21)	50 of 157 (32)	
**Highest level of education**			
High school graduate or less	23 of 95 (24)	20 of 158 (13)	.001
Some college	49 of 95 (52)	66 of 158 (42)	
College graduate	23 of 95 (24)	72 of 158 (46)	
**Annual household income, $^c^**			
<20,000	30 of 88 (34)	33 of 145 (23)	.14
20,000 to <50,000	28 of 88 (32)	49 of 145 (34)	
≥50,000	30 of 88 (34)	63 of 145 (43)	
**Health insurance**			
Private insurance or health maintenance organization	40 of 90 (44)	106 of 156 (68)	.001
Medicaid or Medicare	35 of 90 (39)	42 of 156 (27)	
Other insurance	7 of 90 (8)	4 of 156 (3)	
None	8 of 90 (9)	4 of 156 (3)	
**Has a regular physician**	73 of 95 (77)	146 of 158 (92)	<.001
**Ever goes to physician for a medical checkup**	66 of 95 (69)	139 of 158 (88)	<.001
**Had a routine checkup in the past 12 months**	65 of 95 (68)	128 of 158 (81**)**	.03

a Data were collected in a 1-group pretest–posttest pilot study to assess the feasibility of implementing an intervention led by community health advisors to promote cancer screening (for breast, cervical, colorectal, and prostate cancer) at 9 African American churches in South Los Angeles. All values are numerator and denominator (percentage) unless otherwise indicated.

b Determined by χ^2^ test for categorical variables, 2-sample *t* test for continuous variables.

c Data were missing for 20 participants.

We found no significant differences between the participants who completed the 3-month follow-up survey and noncompleters for residence (South Los Angeles zip codes vs other zip codes, *P* = .56), sex (*P* = .57), or age (*P* = .93). However, participants who completed the 3-month follow-up survey were significantly more likely than noncompleters to report at baseline that they had ever had a colonoscopy (45% vs 28%; *P* = .006), mammogram (96% vs 86%; *P* = .007), or Pap test (96% vs 83%; *P* = .009). Men who completed the 3-month follow-up survey and noncompleters did not differ in ever having had a PSA test (39% vs 31%; *P* = .44) or discussing a PSA test with a physician (18% vs 17%; *P* = .94).

### Potential effect size of the intervention

The worst-case analysis, which included all baseline participants, showed that a substantial proportion of men discussed colorectal cancer screening (31%) and prostate cancer screening (28%) with a physician and a substantial proportion of women discussed colorectal cancer screening (36%), breast cancer screening (43%), and cervical cancer screening (33%) with a physician (Table 4).

**Table 4:** Discussion and Receipt of Cancer Screening During the 3-Month Follow-Up Period Among Participants Who Were Nonadherent at Baseline to ≥1 Cancer Screening Guideline, Los Angeles, 2016–2018^a^

Outcome	As-Reported Analysis^b^		Worst-Case Analysis^c^		
	Men	Women	Men	Women	*P* Value^d^
**Discussed with doctor screening for**					
Colorectal cancer	38 of 95 (40)	76 of 158 (48)	38 of 124 (31)	76 of 214 (36)	.36
Breast cancer	—e	91 of 158 (58)	—e	91 of 214 (43)	—e
Cervical cancer^f^	—e	47 of 102 (46)	—e	47 of 144 (33)	—e
Prostate cancer	35 of 95 (37)	—e	35 of 124 (28)	—e	—e
**Had a . . . (only asked if nonadherent at baseline to . . .)**					
Stool blood test	4 of 62 (6)	23 of 101 (23)	4 of 84 (5)	23 of 142 (16)^g^	.01
Colonoscopy	7 of 62 (11)	14 of 101 (14)	7 of 84 (8)	14 of 142 (10)^g^	.70
Mammogram	—e	27 of 79 (34)	—e	27 of 111 (24)	—e
Pap test^f^	—e	10 of 41 (24)	—e	10 of 60 (17)	—e

Abbreviations: Pap, Papanicolaou.

a Data were collected in a 1-group pretest–posttest pilot study to assess the feasibility of implementing an intervention led by community health advisors to promote cancer screening (for breast, cervical, colorectal, and prostate cancer) at 9 African American churches in South Los Angeles. All values are numerator and denominator (percentage) unless otherwise indicated.

b Includes only those who completed the 3-month follow-up survey (N = 253).

c Includes all baseline participants (N = 338); missing follow-up data were coded as not discussed/not screened.

d χ^2^ test comparing men and women in worst-case analysis.

e Does not apply.

f Only asked of women aged 50 to 65 without hysterectomy.

g Two women reported receipt of both blood test and colonoscopy at follow-up, for a total of 35 of 142 (25%) who received any colorectal cancer screening test during follow-up.

Smaller percentages of participants reported receipt of a screening test. Overall, 13% of men and 25% of women reported receipt of colorectal cancer screening. Among the 84 men who were nonadherent to colorectal cancer screening at baseline, 4 (5%) reported receipt of a stool blood test and 7 (8%) reported receipt of a colonoscopy at 3-month follow-up. Among women who were nonadherent at baseline, 16% (23 of 142) reported receipt of a stool blood test, 10% (14 of 142) receipt of a colonoscopy, 24% (27 of 111) receipt of a mammogram, and 17% (10 of 60) receipt of a Pap test at 3-month follow-up.

### Intervention acceptability

At 3-month follow-up, most participants stated that they would recommend CHA counseling to a friend (94%) and that they would attend a counseling session on another topic if it was offered (83%). Among those who stated that they had discussed cancer screening with a CHA, 91% said that they learned something important from their discussion and 98% felt comfortable asking questions. The following quotes illustrate some of the things participants learned:

- “Early detection can save your life.”
- “I need to take better care of my health.”
- “I never heard of stool blood test; it is easier than colonoscopy.”
- “I learned about increased risk for cancer with age.”
- “Remember to get screened even if you feel healthy.”
- “Getting checked is better than trying to avoid the doctor.”

A CHA from one of the churches stated, “Each person I talked to . . . I felt I was really doing something.” That quote summarizes the sentiments expressed by several CHAs and indicates that the intervention was acceptable to CHAs as well.

## Implications for Public Health

Although CHAs did not systematically collect information on how many people refused to participate in the intervention, they estimated the proportion to be less than 10% of the people they approached. This and the fact that almost half (47%) of African Americans attend religious services at least once per week (32) suggest that cancer screening promotion through church health ministries can potentially reach a large number of community members. Churches like to be inclusive, and CHAs at some of the smaller churches felt it would be difficult to reach their recruitment goals if the study were limited to church members. Because intervention components in our study were primarily directed toward individuals, our study allowed CHAs to recruit people who were not church members. However, if a substantial intervention component is directed toward church activities, church membership and regular attendance would be required for exposure to the intervention.

Although we attempted to keep all study materials simple, several CHAs found it difficult to assess adherence to 3 tests for colorectal cancer screening, each with a different recommendation on frequency. Regular debriefing sessions at each church provided the opportunity to discuss completed assessments and to clarify questions about adherence to national cancer screening guidelines.

CHAs conducted a substantial number of counseling sessions, and records indicated very good implementation of one-on-one education. However, print materials were distributed only as needed (eg, if a participant was not familiar with a screening test), because we did not want to burden participants with unwanted materials. CHAs also had some problems reaching participants by telephone to issue reminders. We learned that many seniors in South Los Angeles scan their incoming calls and do not accept calls from unknown numbers because of widespread telemarketing and fear of scams. Providing the full dose of an intervention (eg, all intervention components) is a challenge in real-life settings. In another study, which offered 3 CHA-led educational workshops to promote cancer screening in African American churches, only 24% of participants attended all 3 workshops, thereby receiving the full dose (10). Because implementation fidelity can affect cancer screening or other outcomes (10), it needs to be carefully monitored and considered when interpreting study outcomes.

Most CHAs were able to conduct one-on-one counseling, but only about one-third of CHAs provided counseling for more than 10 overdue screening tests. Thus, although the burden of serving as a CHA was manageable, only a few CHAs acquired the skills developed by repeat counseling. Overall, implementation required intensive training and monitoring. Although implementation of this intervention was feasible, modifications to simplify and reduce data collection protocols may be required to sustain the effort.

All data were based on self-reports by CHAs and participants, which are susceptible to social desirability bias. Because we lacked a control group, we do not know how many of the screening tests reported at follow-up can be attributed to the intervention. In addition, estimates for potential effect size may have been influenced by selection bias and differential drop-out rates; participants who did not discuss screening or did not obtain a needed test may have been more likely to drop out (ie, defined as not completing the 3-month follow-up survey). Our worst-case analysis attempted to account for some of these limitations. Overall, the potential effect size is similar to that reported by other studies that partnered with African American churches (17,33). Our convenience sample of 9 churches may not be representative of all African American churches in South Los Angeles. In addition, we did not assess maintenance of the intervention beyond the 1-year commitment of each church.

Our study supports the feasibility and acceptability of promoting cancer screening in partnership with health ministries in African American churches. We reported on one of the first studies in African American churches in which CHAs, rather than research team members, assessed screening history and in which CHAs promoted screening for multiple cancers. Our study demonstrates that with training and ongoing technical assistance, CHAs can implement complex research protocols with good fidelity.
